# Racial differences in pathways to care preceding first episode mania or psychosis: a historical cohort prodromal study

**DOI:** 10.3389/fpsyt.2023.1241071

**Published:** 2023-09-04

**Authors:** Manuel Gardea-Resendez, Javier Ortiz-Orendain, Alessandro Miola, Manuel Fuentes Salgado, Mete Ercis, Brandon J. Coombes, Peggy M. Gruhlke, J. Michael Bostwick, Ian Michel, Jennifer L. Vande Voort, Aysegul Ozerdem, Alastair McKean, Mark A. Frye, Monica Taylor-Desir

**Affiliations:** ^1^Department of Psychiatry, Universidad Autónoma de Nuevo León, Monterrey, Mexico; ^2^Department of Psychiatry and Psychology, Mayo Clinic, Rochester, MN, United States; ^3^Department of Psychiatry and Behavioral Sciences, University of New Mexico, Albuquerque, NM, United States; ^4^Department of Neuroscience (DNS), University of Padova, Padua, Italy; ^5^Department of Quantitative Health Sciences, Mayo Clinic, Rochester, MN, United States; ^6^Mayo Clinic Alix School of Medicine, Mayo Clinic, Rochester, MN, United States

**Keywords:** first episode mania, first episode psychosis, prodrome, bipolar disorder, schizophrenia

## Abstract

**Background:**

There is evidence suggesting racial disparities in diagnosis and treatment in bipolar disorder (BD) and schizophrenia (SZ). The purpose of this study is to compare psychiatric diagnoses and psychotropic use preceding a first episode of mania (FEM) or psychosis (FEP) in racially diverse patients.

**Methods:**

Using a comprehensive medical records linkage system (Rochester Epidemiology Project, REP), we retrospectively identified individuals diagnosed with BD or SZ and a documented first episode of mania or psychosis. Illness trajectory before FEP/FEM were characterized as the time from first visit for a mental health complaint to incident case. Pathways to care and clinical events preceding FEP/FEM were compared based on subsequent incident case diagnosis (BD or SZ) and self-reported race (White vs. non-White).

**Results:**

A total of 205 (FEM = 74; FEP = 131) incident cases were identified in the REP. Duration of psychiatric antecedents was significantly shorter in non-White patients, compared to White patients (2.2 ± 4.3 vs. 7.4 ± 6.6 years; *p* < 0.001) with an older age at time of first visit for a mental health complaint (15.7 ± 6.3 vs. 11.1 ± 6.0 years; *p* = 0.005). There were no significant differences by race in FEM pathway to care or age of first seeking mental health. Overall non-White patients had lower rates of psychotropic use.

**Conclusion:**

These data are unable to ascertain reasons for shorter duration of psychiatric antecedents and later age of seeking care, and more broadly first age of initial symptom presentation. If symptoms are confirmed to be earlier than first time seeking care in both groups, it would be important to identify barriers that racial minorities face to access timely psychiatric care and optimize early intervention strategies.

## 1. Introduction

Many patients with bipolar disorder (BD) and schizophrenia (SZ) experience a significant diagnostic confirmation delay as early symptoms are often unrecognized and non-specific with a number of different factors that, on balance, delay treatment initiation (1–3). The heterogeneity of the clinical presentations of mood disorders (i.e., BD and major depression) and SZ, with often-overlapping symptomatology including psychotic symptoms in mania and mood symptoms in schizophrenia, challenge the concept of unique prodromes for each mental illness (4, 5). There is a controversy in the ascertainment of BD and SZ prodromes and delineating time-frame and symptom presentation preceding a first episode of mania (FEM) or psychosis (FEP) may help clarify early disease origins and trajectory of illness (6). There is consensus in the utility of prodromes, if reliably defined, for enhancing early signal detection that would facilitate rapid diagnosis or specific early surveillance of symptoms or symptom progression (7–10). In this line, retrospective assessments of pathways to care, such as mental health service utilization before a first episode, patterns of symptom endorsement and psychotropic use preceding a FEM or FEP could further contribute to the ascertainment of prodromal phases (11). However, efforts to define illness trajectories common to all BD and SZ patients, respectively, are further complicated by factors associated with health disparities and barriers to access healthcare, particularly in racially diverse individuals (12–14).

It has long been recognized that race-related factors, including but not limited to barriers to access health care, play a deleterious role in the timely diagnosis at the initial presentation of BD and SZ (13, 14). Furthermore, it has been consistently reported that non-White patients living with a mood disorder have less use of mental health care services, related, in part, to disparities in social determinants of health, discrimination, science and medical mistrust, inadequate health insurance coverage and lack of clinicians’ cultural competency, among others (15–18). However, provider-related factors such as racial biases and lack of cultural sensitivity have a more pervasive effect in symptom underestimation/overestimation and mischaracterization, increasing the risk of a subsequent misdiagnosis (19). The latter accentuates differences in selection of treatment strategies for BD and SZ, with a severe underutilization in racial minorities of psychotropics, many of which are considered first line options, including less overall use of lithium and mood stabilizing anticonvulsants and higher use to atypical and select typical antipsychotics in BD patients of African ancestry (AA), in comparison to European ancestry and a lower likelihood for Hispanic and Black patients with psychosis to receive atypical antipsychotics (16, 20, 21).

Recognizing the known racial disparities in access to care for BD and SZ, in this study we sought to retrospectively review pathways to care and longitudinal patterns of mental healthcare utilization, psychiatric diagnoses and psychotropic treatments in White and non-White individuals from 27 counties in Minnesota who progressed to meet diagnostic criteria for BD or SZ.

## 2. Methods

### 2.1. Data source and study population

Subjects were identified through the Rochester Epidemiology Project (REP) which is an integrated comprehensive medical record utilized thus far for more than 2000 observational studies of acute and chronic disease incidence and outcomes, including bipolar disorder, in a defined population of Olmsted County, Minnesota and 26 additional surrounding counties. Findings from studies using the REP have shown to be extrapolated to other populations in the United States and globally (22). This population-based cohort addresses limitations observed in clinical series, with referral bias, and lack of complete case ascertainment in administrative data bases. Previous studies on schizophrenia and bipolar disorder have been performed using REP, offering long-term perspectives of cohorts with confirmed psychiatric disorders (23–26).

Inclusion criteria for the initial search were (a) patients born after 1985 who (b) diagnosed with bipolar disorder (types I or II) or schizophrenia spectrum disorders, including schizoaffective disorder, (c) between 2000 and 2019 and (d) resided in Olmsted County and surrounding counties on the date of the diagnostic code assignment. The initial search yielded 1,335 subjects who were selected for further manual screening by two psychiatrists (MG and JO-O) for diagnostic confirmation, based on DSM criteria, and for identification of individuals with a recorded first episode of mania or psychosis (FEM or FEP, respectively) in their medical charts. Disagreements in case ascertainment were solved by a panel of three senior psychiatrists (AMc, AO, and MAF). The latter search identified a total of 205 (SZ = 131; BD = 74) with a first episode recorded and were further analyzed for characterization of the illness trajectory and included for this study, while those without a clearly recorded first episode were excluded from the study. Detailed information about the specifics of the search strategy, including specific diagnostic codes used for BD and SZ, coding systems, case ascertainment, extracted data and study population has been previously published (27).

This study received approval from the institutional review boards (IRBs) of Mayo Clinic and Olmsted Medical Center, who provided a Health Insurance Portability and Accountability Act (HIPAA) waiver, in line with state, federal, and international recommendations. Due to the latter, written informed consent was not required for passive medical record review in the REP (28).

### 2.2. Classification of race and ethnicity

Within the REP, race and ethnicity are determined by patient self-report, and listed categories are based on the standardized classification of the National Institutes of Health (NIH) to promote uniformity of data on race and ethnicity, including an additional option for patients who refused to provide these data and one for those who self-identified with another or more than one race (29). Categories for race include American Indian or Alaska Native, Asian or Asian American (including East Asian), Black/African/African American, Native Hawaiian or Other Pacific Islander, White, Other and Unknown (i.e., unavailable data, patient refusal to specify). Categories for ethnicity include Hispanic/Latino, Not Hispanic/Latino and Unknown.

The racial and ethnic composition of the population in the REP is predominantly White but known to be representative of Minnesota and the Upper Midwest and to be similar to the overall composition of the United States’ population (28). Hence, to account for the underrepresentation of some non-White racial and ethnic groups, for this study, participants were further categorized into two broad categories: White (W), for patients of self-reporting as White and not Hispanic/Latino ethnicity, and non-White (nW), which included individuals who self-identified with any race other than White, including those who did not specify their race and/or Hispanic/Latino ethnicity.

### 2.3. Categorization of illness trajectory based on clinical encounters

#### 2.3.1. Clinical characteristics

We searched the REP and associated medical records to identify distinct types of early life traumatic experiences (e.g., physical or emotional abuse) and diagnostic codes of common child and adolescent psychiatric disorders. Assessment of substance use was defined as the utilization of any licit or illicit substance without necessarily constituting a substance use disorder (i.e., experimental use). Exposure to psychotropic medications prior to an incident case was collected through review of clinical notes and prescription registry in the REP, and medications were classified by psychotropic class (e.g., antipsychotics, antidepressants, etc.) and specific drug name.

#### 2.3.2. Pathways to care and prodrome

The pathway to care start point was defined as the first encounter with a health care provider on which an International Classification of Disease (ICD) code for a psychiatric diagnosis or complaint was added to the patient’s chart, while the first episode, or endpoint, was defined as the clinical encounter in which the patient was first observed to have symptoms of psychosis or mania and received an associated diagnosis, as determined by a clinician’s assessment. The first contact with REP was defined as the first interaction with a health provider in the Olmsted County area (i.e., Mayo Clinic, Olmsted Medical Center) for any health concern.

### 2.4. Statistical analysis

We first compared sociodemographic and clinical variables, and rates of psychotropic prescriptions between White and non-White individuals with a known diagnosis of BD or SZ using chi-square tests (*χ*^2^) for categorical measures and two-sample *t*-tests for continuous measures, respectively. We then tested for differences in prodrome duration (as defined by age at first visit for a mental health complaint to age at incident), age at first medical visit and first visit for a mental health complaint, and age of first psychiatric hospitalization between the same groups using ANOVA (two-sample *t*-tests) or Kruskal–Wallis non-parametric ANOVA based on normality assumption. Statistical analyses were performed using Jamovi.^1^

## 3. Results

### 3.1. Demographic composition of the cohort

**Figure 1:**
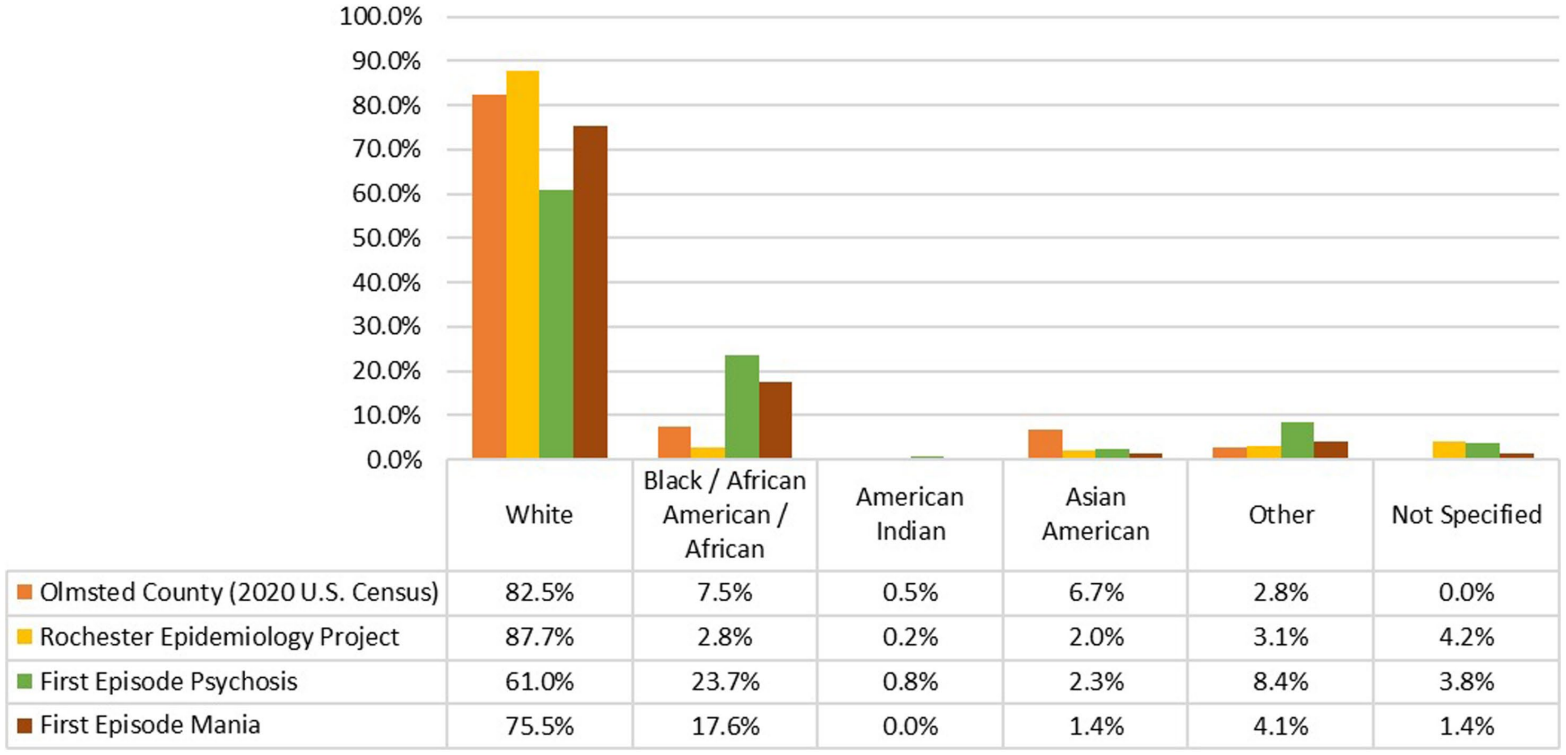
Comparison of the racial composition of population from Olmsted County based on the 2020 U.S. Census (30), population captured in the expanded Rochester Epidemiology Project (E-REP) (28), individuals included in this study and identified in the REP with a first episode of psychosis and, a first episode of mania.

We identified 205 subjects, 74 with a first episode of mania (FEM) and 131 with a first episode of psychosis (FEP); both samples were comprised mainly of men (FEM = 60.8%; FEP = 80.9%). The self-reported racial composition of the FEM group was 75.7% White, 17.6% African/African American/Black, 1.4% of Asian/Asian American, 3% as Other and 1.4% did not specified their racial background, whereas in the FEP group, 61.1% of the participants self-identified as White, 23.7% were of African/African American/Black, 8.4% as Other race, 2.3% of Asian/Asian American, 0.8% Indigenous American and 3.8% that did not specify their race (Figure 1). Of note, all of the subjects in the “Other” subgroup self-identified as Hispanic/Latino.

Within the FEP group, 51% of the non-White participants (*n* = 26) were born outside of the United States (48% in Somalia, 16% in Sudan, 16% in Mexico, 8% in Ethiopia, 8% in Kenya and 4% in Saudi Arabia). Based on further chart review, within the foreign-born patients, 50% voluntarily immigrated to the U.S. and 26.9% arrived as refugees (forced migration), while no information regarding immigration status was found for the remaining 23.1%. Only four participants with FEM (22.2% of the non-White group) were foreign-born (Somalia), three of which arrived as refugees and one as voluntary immigrant. For both FEP and FEM, mean age (SD) at arrival to the United States of those with available data (*n* = 28) was 10.3 (6.5) years. In this line, childhood trauma associated with war and terrorism was significantly more common in non-White individuals with a FEP (9.8% vs. 0%; *p* = 0.004).

### 3.2. Psychiatric morbidity before a first episode

Table 1 shows the clinical features preceding a FEM or FEP divided by White and non-White participants. In general, there was increased morbidity of psychiatric diagnoses preceding a first episode of psychosis or mania in White patients. A diagnosis of attention-deficit/hyperactivity disorder in non-White individuals with a FEP was much less common when compared to White individuals (15.7% vs. 52.5%; *p* < 0.001). This was not observed for FEM. The same trend was observed in autism spectrum disorder before a FEP (0% vs. 8.8%; *p* = 0.03). No large differences in conduct disorders and oppositional defiant disorder were observed in the two cohorts.

**Table 1:** Clinical characteristics preceding a first episode mania (FEM) or psychosis (FEP) by racial categories (White vs. non-White patients).

	First episode mania/bipolar disorder (*n* = 74)			First episode psychosis/schizophrenia (*n* = 131)		
	White (*n* = 56)	Non-White (*n* = 18)	*p*-value	White (*n* = 80)	Non-White (*n* = 51)	*p*-value
Age at first episode, mean (SD)	21.8 (3.5)	20.1 (3.7)	0.11	20.5 (4.1)	20.3 (3.5)	0.83
Sex, male	34 (60.7%)	11 (61.1%)	0.98	63 (78.8%)	43 (84.3%)	0.43
*Place of birth*						
U.S.-born	56 (100%)	14 (77.8%)	<0.001	78 (97.5%)	25 (49%)	<0.001
Foreign-born	0	4 (22.2%)		2 (2.5%)	26 (51%)	
*History of childhood trauma*						
Sexual abuse	11 (19.6%)	3 (16.7%)	0.78	12 (15.0%)	3 (5.9%)	0.11
Physical abuse or neglect	13 (23.2%)	3 (16.7%)	0.56	18 (22.5%)	3 (5.9%)	0.01
Emotional abuse or neglect	13 (23.2%)	3 (16.7%)	0.56	16 (20.0%)	2 (3.9%)	0.01
Family dysfunction	21 (37.5%)	6 (33.3%)	0.75	16 (20.0%)	5 (9.8%)	0.12
Bullying and/or cyberbullying	4 (7.1%)	0 (0.0%)	0.24	10 (12.5%)	2 (3.9%)	0.09
Separation from parent or caregiver	4 (7.1%)	4 (22.2%)	0.07	10 (12.5%)	7 (13.7%)	0.84
War and/or terrorism	0	2 (11.1%)	0.01	0 (0.0%)	5 (9.8%)	0.004
*Psychiatric diagnoses before the FE*						
ADHD	17 (30.4%)	6 (33.3%)	0.81	42 (52.5%)	8 (15.7%)	<0.001
Intellectual disability	0	2 (11.1%)	0.01	7 (8.8%)	2 (3.9%)	0.29
Autism spectrum disorder	0	0	—	7 (8.8%)	0	0.03
Conduct disorder	6 (10.7%)	1 (5.6%)	0.52	11 (13.8%)	4 (7.8%)	0.30
Oppositional defiant disorder	4 (7.1%)	2 (11.1%)	0.6	11 (13.8%)	2 (3.9%)	0.07
Depressive disorders	40 (71.4%)	7 (38.9%)	0.01	48 (60.0%)	14 (27.5%)	<0.001
Anxiety disorders	27 (48.2%)	4 (22.2%)	0.05	32 (40.0%)	10 (19.6%)	0.02
Adjustment disorders	22 (39.3%)	5 (27.8%)	0.38	20 (25.0%)	7 (13.7%)	0.12
PTSD	4 (7.1%)	3 (16.7%)	0.23	5 (6.3%)	5 (9.8%)	0.50
*Substance use before FE*						
Alcohol	32 (57.1%)	6 (33.3%)	0.08	41 (51.2%)	14 (27.5%)	0.007
Tobacco	26 (46.4%)	5 (27.8%)	0.16	28 (35.0%)	19 (37.3%)	0.79
Stimulants	7 (12.5%)	4 (22.2%)	0.31	17 (21.3%)	5 (9.8%)	0.09
Cannabis	37 (66.1%)	11 (61.1%)	0.70	55 (68.8%)	35 (68.6%)	0.98
Cocaine	3 (5.4%)	3 (16.7%)	0.13	11 (13.8%)	2 (3.9%)	0.07
Hallucinogens	3 (5.4%)	1 (5.6%)	1.0	16 (20.0%)	2 (3.9%)	0.01
Hypnotics	6 (10.7%)	2 (11.1%)	1.0	9 (11.3%)	1 (2.0%)	0.05
Opioids	5 (8.9%)	2 (11.1%)	1.0	10 (12.5%)	1 (2.0%)	0.03
*Psychotropic prescriptions before FE*						
Any psychotropic	40 (71.4%)	8 (44.4%)	0.04	60 (75.0%)	18 (35.3%)	<0.001
Antidepressants	36 (64.3%)	5 (27.8%)	0.01	47 (58.8%)	14 (27.5%)	<0.001
Mood stabilizers	4 (7.1%)	0 (0.0%)	0.24	11 (13.8%)	3 (5.9%)	0.155
Antipsychotics	6 (10.7%)	1 (5.6%)	0.52	24 (30.0%)	3 (5.9%)	<0.001
Stimulants	12 (21.4%)	5 (27.8%)	0.58	35 (43.8%)	7 (13.7%)	<0.001

While depressive disorders were very frequent in both FEP and FEM, non-White patients were less frequently diagnosed with depression (FEP: 27.5% vs. 60%; *p* < 0.001; FEM: 38.9% vs. 71.4%; *p* = 0.01). A similar trend was seen in anxiety disorders in both cohorts (FEP: 19.6% vs. 40%; *p* = 0.02; FEM: 22.2% vs. 48.2%; *p* = 0.05).

Within the FEP group, physical and emotional abuse were significantly more common in White individuals, while, as mentioned previously, trauma related to war and terrorism was more common in non-White individuals from both cohorts. Nonetheless, rates of PTSD diagnosis did not significantly differ among groups albeit this diagnosis had a low prevalence prior to FEP/FEM.

With regards to substance use before a first episode, White FEP patients had a significantly higher prevalence of alcohol (51.2% vs. 27.5%; *p* = 0.007), hallucinogens (20% vs. 3.9%; *p* = 0.01), hypnotics (11.3% vs. 2%; *p* = 0.05) and opioid use (12% vs. 2%; *p* = 0.03). While limited by a lower sample size, In FEM, a similar trend was seen for alcohol use, while the opposite was seen for the rest of the assessed substances, where substance use was more common in non-White individuals; these findings, however, were not significant and limited by a lower sample size.

### 3.3. Patterns of psychotropic use

Overall, psychotropic prescriptions before a first episode were significantly more common in White patients who developed a FEM or a FEP than in non-White patients (FEM 71.4% vs. 44.4%, *p* = 0.04; FEP 75% vs. 35.3%, *p* < 0.001). After stratifying by psychotropic groups, differences between racial categories were still statistically significant, particularly in FEP. Antidepressant use before a FEM was significantly more common in White patients (64.3% vs. 27.8%, *p* = 0.01); the same prescription trend was observed in FEP (58.8% vs. 27.5%, *p* < 0.001). Antipsychotic use before a FEP was also significantly more common in White individuals (30% vs. 5.9%, p < 0.001) and so were stimulant prescriptions (43.8% vs. 13.7%, *p* < 0.001); no significant differences were observed in antipsychotic and stimulant use before a FEM. Mood stabilizer use before a first episode was uncommon in all groups.

### 3.4. Pathways to mental health care and illness trajectories

Table 2 shows the illness trajectories preceding a FEM or FEM in White and non-White patients. Non-White patients from both cohorts (FEM and FEP), when compared to White patients, began receiving health care at Olmsted County (as defined by age of first contact with REP) at a significantly older age (BD: 7.4 ± 7.3 vs. 4.5 ± 8.6; *p* = 0.01; SZ: 11.5 ± 7.4 VS. 3.3 ± 5.9; *p* < 0.001). Age at the time of a first episode did not vary between groups in both patients with psychosis and with mania and was consistent with the ages of onset reported in the literature (4). However, the overall duration of the prodrome (i.e., time from first visit for a mental health complaint to incident case) was significantly shorter in non-White individuals with a FEP, when compared to White individuals (2.2 ± 4.3 vs. 7.4 ± 6.6 years; *p* < 0.001); no significant differences were observed in the prodrome duration for bipolar patients. Similarly, non-White patients who further developed a FEP had their first visit for a psychiatric complaint (prodrome onset) at a significantly older age than White patients (15.7 ± 6.3 vs. 11.1 ± 6.0 years; *p* = 0.005). No significant differences were observed in the age of first psychiatric hospitalization for both cohorts (BD and SZ). However, hospitalization before first episode was uncommon in both cohorts (BD = 17; SZ = 21).

**Table 2:** Illness trajectory preceding a first episode mania (FEM) or psychosis (FEP) by racial/ethnic categories (White vs. non-White patients).

Illness trajectory variable (in years)			Mean (SD)	*F*	*p*-value
Duration of psychiatric antecedents	FEM	Non-White (*n* = 18)	5.5 (6.2)	0.07	0.8
		White (*n* = 56)	5.5 (6.1)		
	FEP	Non-White (*n* = 51)	2.2 (4.3)	22.26	<0.001
		White (*n* = 80)	7.4 (6.6)		
Age of first contact with REP	FEM	Non-White (*n* = 18)	7.4 (7.3)	2.76	0.01
		White (*n* = 56)	4.5 (8.6)		
	FEP	Non-White (*n* = 51)	11.5 (7.4)	39.72	<0.001
		White (*n* = 80)	3.3 (5.9)		
Age of first visit for a mental health complaint	FEM	Non-White (*n* = 12)	12.1 (5.6)	1.54	0.23
		White (*n* = 43)	14.3 (5.5)		
	FEP	Non-White (*n* = 24)	15.7 (6.3)	7.90	0.005
		White (*n* = 62)	11.1 (6.0)		
Age of first psychiatric hospitalization	FEM	Non-White (*n* = 2)	15.3 (4.5)	0.19	0.72
		White (*n* = 15)	16.9 (4.4)		
	FEP	Non-White (*n* = 3)	14.9 (0.5)	0.5	0.48
		White (*n* = 18)	16.5 (3.7)		

## 4. Discussion

To our knowledge, this is the first study using representative data from a community sample to compare racial differences in pathways to care, conceptualized from clinical events and psychiatric care (i.e., diagnoses, psychotropic use, mental health service utilization) preceding a first episode of mania or psychosis. In a U.S. patient database built upon a medical records-linkage system, non-White patients with a known diagnosis of schizophrenia, when compared to White patients, had a significantly shorter duration of the pathways to care, as defined by the time from the first visit for a mental health complaint to the incident case (FEP). This shortened duration appears to be driven, among other factors, by a first mental health-related clinical contact at a significantly older age than White patients. As a potential consequence of this delayed initiation in psychiatric care, particularly in SZ, prodromal phases in non-White patients were characterized by fewer psychiatric diagnoses and lower rates of psychotropic utilization in comparison to White patients. While these some of these differences were not statistically significant in patients with bipolar disorder, the trends were somewhat similar. Furthermore, White patients from both cohorts had their first contact with the health care system in Olmsted County at a significantly earlier age than non-White patients.

The present study has several strengths. First, by focusing on the prodromal phases of bipolar disorder and schizophrenia in racially diverse groups specifically, we addressed an important gap in the literature, offering insights into the pathways to care and clinical experiences that precede an incident case of mania or psychosis in patients of racially diverse backgrounds, enabling a better of understanding of the lived experiences and barriers that minoritized individuals face when seeking mental health care. Second, all cases were individually assessed and had the diagnosis and date of first episode manually confirmed by psychiatrist chart review providing higher diagnostic validity and reliability. In this line, the use of REP resources enabled an extensive follow-up from first contact with the local healthcare providers until incident case of mania or psychosis, allowing for a retrospective assessment of a well-defined population-based cohort while addressing the risk of recall bias (23).

Our finding of a significantly shorter duration of the pathways to care in non-White patients with a FEP, rather than corresponding to a race-specific variation of symptom presentation, may suggest that racially diverse patients experience an aversive pathway to care (i.e., health mistrust, lack of insurance coverage, etc.) that leads to a subsequent delay in the first contact with health services for psychiatric complaints. While, at first sight, the lack of variations at age of first episode in both BD or SZ might suggest that the illness onset was not impacted by the delayed initiation of mental health care, further assessment of the symptom severity of the first episode and the clinical setting (emergency room, outpatient/inpatient clinics, etc.) where it was diagnosed may provide a more comprehensive and accurate assessment of the impact of delayed care. Restricted access to care, which disproportionately impacts racial and ethnic minorities, inevitably prolongs the duration of untreated illness, which has been associated with worse symptomatic and functional outcomes and subsequent illness trajectories of bipolar disorder and schizophrenia (31, 32). Barriers to access timely mental healthcare combined with other racial biases that non-White patients with severe mental illnesses experience can lead to more severe clinical presentations that can further increase the likelihood of an initial misdiagnosis (17). In this line, earlier initiation of psychiatric care and more frequent use of health services may account for the higher prevalence of psychiatric disorders preceding a first episode in White patients seen in our study, whereas the opposite was observed in non-White patients. This hypothesis is further supported by findings from a study conducted using a commercial claims database and focused on health care use before a FEP, which reported lower behavioral health care use rates and outpatient visits in Hispanic and Black patients, when compared to White patients. Similarly, in this study, a lower likelihood of receiving a comorbid psychiatric disorder before a FEP was observed in Black and Hispanic patients (12). Although our study design does not allow the establishment of causality inferences, the coincidence of our findings with the existing body of evidence supports the notion that non-White patients may experience barriers to access timely psychiatric care before an incident case of mania or psychosis resulting in fewer psychiatric diagnoses and lower psychotropic use (11, 17, 33).

Psychotropic medication use before a first episode was significantly lower in racially diverse patients from both of our study groups. This finding was consistent across all psychotropic categories except mood stabilizers. In particular, antidepressant prescriptions were significantly lower in non-White patients which is particularly relevant considering the high rates of depression preceding an index (hypo)manic episode and the frequency of a depressive index episode (over 50% of the cases) (34). However, in our cohort, diagnosis of depressive disorders was significantly more common in White patients from both groups. Considering that the age of FEM did not vary significantly between racial groups despite the differential exposure to antidepressants, there is value in further analyzing if early exposure to antidepressants, or lack thereof, has an impact, either preventive or precipitating, in the onset of a first (hypo)manic episode. A similar assessment focused on the controversy of risk of conversion to psychosis in high-risk subjects exposed to antipsychotics might also provide further data on the disease-modifying effect of neuroleptics (35, 36). Our finding of lower psychotropic use before a first episode aligns with previous studies reporting that among youths with psychiatric complaints, those from racial minority groups, particularly individuals who self-identify as Black, Latino or Asian, are less likely than White individuals to be prescribed psychotropic medications when impaired (37–39). This trend towards lesser prescription of specific psychotropics in non-White adolescents with BD and SZ tends to persist and exacerbate in adulthood, even in well-established illness, were, as previously mentioned, lower rates of lithium and antiepileptic mood stabilizers utilization in African American individuals with BD have been reported, despite lithium being a gold-standard treatment, and persistently higher rates of first-generation antipsychotics in BD and SZ, at the expense of lower atypical antipsychotic use (16, 40, 41). These differences in psychotropic use occur in spite of the increasing evidence supporting their clinical applications and higher tolerability, deviating from psychotropic prescription trends and omitting FDA approvals and clinical guidelines (42). While multifactorial, the origins of these differential prescription patterns have been linked to race-related disparities, including clinicians’ implicit ethnic/racial biases, structural racism, misattribution of sociocultural expressions and behaviors as manifestations of psychopathology arising from the lack of cultural competency, and it’s reasonable to hypothesize that this affects individuals seeking help during prodromal phases of BD and SZ in a similar fashion (11, 43).

While our study offers insights into potential health disparities in non-White patients before a first episode of mania or psychosis, this study has several limitations that need to be considered. In merging individuals of other than White racial backgrounds into one large group (non-White patients) there is a risk of overlooking the unique experiences of each racial group. This decision was made in order to address the relatively small sample size of certain racial groups, a consequence of the racial composition of Olmsted County. Because some patients in the different cohorts were not born in Olmsted County, Minnesota and moved in later, this might have altered the age of first contact with the regional health system. The retrospective design of this study entails several risks of bias including selection bias; however, the REP addresses limitations known to administrative registry-based databases, which are frequently passively fed with diagnoses and procedures, by including longitudinal comprehensive medical information from individual medical records (44). Third, the lack of a standardized definition for prodromes in, both, bipolar disorder and schizophrenia represents a significant challenge for studies assessing clinical events preceding an incident case; we sought to reduce the risk of recall bias by relying on clinical encounters to conceptualize pathways to care, however this approach might omit early mild or subclinical symptoms preceding a first episode. Finally, while 51% of our non-White FEP patients were born outside of the U.S., and recognizing that migration and forced displacement are well-known social risk factors for psychosis and mood disorders and that immigrants and refugees are at higher risk of marginalization and cumulative trauma, among other environmental stressors, we did not conducted a detailed assessment of this cases (i.e., country of birth, age at migration, type of displacement, etc.) that might have helped examine the ways in which risk of severe mental illness intersects with race and migration (45, 46). Our findings suggest race differences in the pathways to care and patterns of psychotropic utilization preceding a first episode of mania or psychosis, specifically an overall lower use of health services and psychotropic medications in non-White patients. While in our study we did not examine the drivers of this delayed treatment initiation in non-White patients, we are aware that, in the U.S., racial minorities with mental health illnesses experience systemic and structural barriers that make timely and affordable access to healthcare and behavioral healthcare difficult, which, in consequence increases the duration of untreated illness and the likelihood of a worse course of illness and adverse outcomes (11, 18). Hence, in the conceptualization of prodromal phases for BD and SZ, it may prove not only advantageous but necessary to integrate racial disparities in healthcare as social risk markers that might exacerbate the diathesis for these disorders and obstruct the identification of at-risk subjects or early-onset disease. Future studies mapping prodromal trajectories of bipolar disorder and schizophrenia must further explore the cause of these racial differences in order to enable the recognition, and subsequent correction, of the adverse lived experiences and health disparities that racial and ethnic minorities face when seeking mental health care.

## Data availability statement

The data analyzed in this study is subject to the following licenses/restrictions: datasets are not available in order to protect patient confidentiality. Requests to access these datasets should be directed to gardearesendez.manuelandres@mayo.edu.

## Ethics statement

The studies involving humans were approved by Mayo Clinic Institutional Review Board, Olmstead Medical Center Institutional Review Board. The studies were conducted in accordance with the local legislation and institutional requirements. Written informed consent for participation was not required from the participants or the participants’ legal guardians/next of kin in accordance with the national legislation and institutional requirements.

## Author contributions

MG-R, AMi, MAF, and JO-O: writing and original draft preparation. MG-R, JO-O, AMi, MFS, ME, BC, PG, MB, IM, JV, AO, AMc, MAF, and MT-D: critical revision of the article contributing with ideas related to their area of expertise. MG-R, JO-O, and PG: data collection. AMi: statistical analysis. MG-R, AMi, BC, JV, JO-O, MAF, MB, MT-D: analysis and interpretation. MG-R, MAF, and BC: contribution to conceptualization. All authors contributed to the article and approved the submitted version.

## Funding

This study was supported, in part, by the Luther Automotive Foundation and Mayo Foundation; neither had a role in the design, conduct, analysis, or submission of the study. This study was also made possible using the resources of the Rochester Epidemiology Project, which is supported by the National Institute on Aging of the National Institutes of Health under Award Number R01AG034676. The content is solely the responsibility of the authors and does not necessarily represent the official views of the National Institutes of Health.

## Conflict of interest

The authors declare that the research was conducted in the absence of any commercial or financial relationships that could be construed as a potential conflict of interest.

## Publisher’s note

All claims expressed in this article are solely those of the authors and do not necessarily represent those of their affiliated organizations, or those of the publisher, the editors and the reviewers. Any product that may be evaluated in this article, or claim that may be made by its manufacturer, is not guaranteed or endorsed by the publisher.

## Footnotes

^1^https://www.jamovi.org/
